# Enhancing performance of Parallel Hybrid Electric Vehicles using Powell's Artificial Bee Colony method

**DOI:** 10.1016/j.heliyon.2025.e42325

**Published:** 2025-01-29

**Authors:** S.N. Shivappriya, T. Gowrishankar, Gabriel Stoian, J. Anitha, D Jude Hemanth

**Affiliations:** a Department of ECE, Kumaraguru College of Technology, Coimbatore, India; b Bosch Global Software Technologies, Coimbatore, India; c Department of Informatics, University of Craiova, Craiova, Romania; d Department of ECE, Karunya Institute of Technology and Sciences, Coimbatore, India

**Keywords:** Hybrid electric Vehicle (HEV), Powell's artificial bee colony optimization approach, Fuel efficiency, State of charge, Emissions, Energy efficiency

## Abstract

Hybrid Electric Vehicles (HEVs) demonstrate superior fuel efficiency and reduced emissions in comparison to conventional vehicles. To further enhance the HEV performance, Powell's based Artificial Bee Colony (ABC) heuristic approach is used. Powell's ABC focuses on the improved local search ability and increased speed of convergence. The multi parameter optimization approach with the PNGV constraints for the four differently weighted objective function parameters, the experiments were carried out for most generally used driving cycles FTP, ECE-EUDC and UDDS. Compared with the initial values, the proposed approach gives the improvement in the fuel efficiency by 10.03 % and the emissions are reduced to a maximum of 18.4 % and improved overall vehicle efficiency is 11.1 % for the ECE-EUDC driving cycle. For the UDDS driving cycle, fuel efficiency can be improved by 18.2 % and the emissions are reduced to a maximum of 43.24 %, improved overall vehicle efficiency 10.1 %. For FTP driving cycle fuel economy by 39.98 % and the emissions are reduced to a maximum of 43.75 %, improved overall vehicle energy efficiency up to 11.6 %. The findings indicate that Powell's ABC approach achieves faster convergence to a notably more precise final solution across various typical driving cycles compared to conventional methods.

## 1 Introduction

Researchers are concentrating on Hybrid Electric Vehicle (HEV), because of the global crisis on fossil fuel resources. In HEV, the benefits of the electric energy source and Internal Combustion Engine (ICE) are combined. This is the best solution with the existing fossil fuel resources. To achieve this, vehicle design should be analyzed to achieve appropriate component size and control strategy with still achieving the on-road vehicle performance. While designing the HEV system, the following points must be considered such as key power sources for the power train system, the energy flow management system. With the proper power flow and control strategy-based optimization technique the vehicle parameters are to be tuned. This results in the improved fuel efficiency, emissions, and vehicle efficiency of the HEVs. With the Advanced Vehicle Simulator (ADVISOR) tool vehicle designing is done and the Mathematics Laboratory (MATLAB) based Pheromone Based Approach (PBA) gives better performance for the following three different important driving cycles: European Extra Urban Drive Cycle (ECE-EUDC), Urban Dynamometer Driving Schedule (UDDS), Federal Test Procedure (FTP). The optimization approach faces the difficulty of exploration and exploitation during the search for the best solution.

Krause, J [1] shown good fuel saving with cost optimal CO2 emission reduction for light duty vehicle. These models allow for the construction of cost curves, identification of cost-effective CO2 emission reduction distributions across the various powertrains and fleet segments, and calculation of additional manufacturing costs, fuel and energy savings, as well as total costs or savings resulting from different scenarios. This work focuses only on CO2 emission. V.T.Long, and N.V.Nhan [2] has shown how the FC and emissions are reduced by maintaining the Partnership for a New Generation of Vehicles (PNGV) constraints, the limits are available in the referenced paper.

Ardashir Mohammadzadeh [3] Introduced a brand-new interval type-3 fuzzy system. Compared to the Traditional Type-2 Fuzzy System (T2FS), it has better approximation capabilities. The fuzzy system's parameters are adjusted using an online stable fractional-order learning algorithm. The rule-based system faces low fuel economy and different cycle needs different control parameters. An Artificial Neural Network (ANN) can be trained using the dataset that Carla et al. [4] created using dynamic programming. It can determine the best driving profile-related performance condition and aid in creating rules for real-time management. Prior knowledge of driving cycle and high computation is required. Pre-processing of the data is needed before fed into the neural network model. Mehrdad Ehsani et al. [5] discussed the problems and potential of future research in Electric Vehicle (EV)/Hybrid Electric Vehicle (HEV).

P.Juan Torreglosa [6] did a survey on Improvements of Energy Management Systems for Hybrid Electric Vehicles, this work gave an analysis of HEV and mentioned the hybridization of vehicles is a feasible solution towards the challenge of the reduction of emissions related to road transport all over the world. Sun C [7] used velocity forecast adaptive Equivalent Consumption Minimization Strategies (ECMS) for improved fuel efficiency and provides stable battery State Of Charge (SOC) path. In this emissions and vehicle dynamics optimization were not considered critically. C.Samanta [8] used Particle Swarm Optimization (PSO) for the energy management in HEV using multi objectives and random search approaches. This method had high consuming time and trapping to suboptimal solution, which prevented it from attaining global solution.

Gujarathi [9] analyzed the Parallel HEV with the hybrid optimization approach of ABC and Grey wolf optimization approach for reducing complexity and computational load by providing balance between the exploitation and exploration. Computational speed can be improved, convergence into local optima is possible. Gowrishankar [10] compared the Basic Artificial Bee Colony BABC algorithm for the three different driving cycles and proposed the modified Artificial Bee Colony algorithm with the improved objective function. The basic ABC has the problem of being good at exploration but poor at exploitation, causing the algorithm to become stranded in local optima and may fail to achieve the global optimal solution. In 2011 Serrao [11] did an analysis on three approaches for energy optimization namely Pontryagin's Minimum Principle (PMP), Equivalent Consumption Minimization Strategy (ECMS) and Dynamic Programming (DP). In the following years, these methods served as the benchmark. This is a complex mathematical modelling approach and requires approximation to reduce the computation burden.

The authors Huang Y [12] and Huang Y [13] examined the EMS with two methods: Model Predictive Control (MPC)-based ones and integrated ones by simultaneously optimizing component size and power management parameters. Özgür Yeniay [14] explained about the penalty functions for the constrained optimization problems. Belen et al. [15], described about the derivative free Powell's optimization technique, with the unrestricted optimization problem. To create a new trial point, a quadratic interpolation model of the goal function is built around the present iteration, throughout the entire procedure, a trust-region framework is used. Marwa Ben Ali, Ghada Boukettaya [16] developed a comparative study between various offline optimization techniques, to achieve an ideal power split between the Internal Combustion Engine (ICE) and the Electric Motor (EM), primarily in the hybrid propulsion mode. Tran et al. [17], evaluated the various powertrain topologies and systems of (plug-in) hybrid electric vehicles and full-electric vehicles.

Fredrikbaberg & Elliotdahl [18] carried out the statistical analysis for the main elements, including advantages and disadvantages, of the most prevalent types of energy management systems utilized in HEVs. Additionally, from the perspective of control theory, links and improvement potential among various energy management systems are shown. However, this study hasn't provided any fundamental designs as examples or significance for improved vehicle design or output for various energy management systems. Zhang et al. (2015) have thoroughly examined the various bibliometric-based HEV energy management solutions and suggest that driving cycle recognition could aid in more effective HEV energy management. The Dynamic Programming (DP) method solves discrete multi-stage decision problems by selecting a decision from a small set of decision variables at each time step based on the optimization criterion [19].

From a mathematical perspective, Equivalently PMP, as presented by Namwook et al. (2011) [20], described the worldwide optimality of the HEV fuel optimization principle with given plausible assumptions. It also explains how the tight connection between the optimal-control-theoretic idea of PMP and the Equivalent Consumption Minimization Strategy (ECMS) leads to ECMS's optimality. Edward D. Tate Stephen P. Boyd (1998) [21] developed a linear programme to solve the problem of determining the minimal fuel consumption for a certain hybrid propulsion system. To determine the absolute performance limit for a series hybrid propulsion system architecture irrespective of any control law, this challenge has been solved. This outcome has been used to determine component requirements and assess the effectiveness of control laws.

GA has overcome the shortcomings of gradient-based optimization methods that require calculating the derivative of the objective function, making it appropriate for this nonlinear optimization issue [22]. GA has eliminated the disadvantages of gradient-based optimization approaches, which require calculating the objective function's derivative, making it suitable for this nonlinear optimization problem. As per the literature review, a comparison of previous works was shown in Table 1. Among the different heuristic approach the Powells ABC is robust to noise and uncertainty in the objective function, making it suitable for real-world problems where data may be incomplete or imperfect. The algorithm scales well with the problem size, maintaining performance even as the number of variables increases, which is beneficial for complex optimization tasks. The algorithm is flexible, relatively simple to implement and requires fewer parameters to tune compared to other optimization techniques, making it accessible for practitioners. Powell's approach is integrated into ABC as a local search tool to improve the algorithm's exploitation. The approach minimises the function by doing a bidirectional search along each search vector in turn. The new position is then represented as a linear combination of the search vectors. The new displacement vector becomes a new search vector. As a result, ABC and Powell's technique offer complimentary advantages, and the proposed algorithm may result in a faster and more reliable method.

**Table 1:** Literature review comparison.

References	Method	Scope of the paper	Parameters not considered
Long & Nhan(2014) [2]	Pheromone-based Bee's Algorithm (PBA)	Fuel economy, emission and vehicle key components size reduction	The optimization strategy that faces the challenge of exploration and exploitation during the search for the optimal solution.
C.Samanta [8]	Particle Swarm Optimization (PSO)	Energy management in HEV using multi objectives and random search approaches	This method had high consuming time and trapping to suboptimal solution, which could prevent it from attaining global solution.
Gujarathi [9]	Hybrid optimization approach of ABC and Grey wolf optimization approach	Reducing complexity and computational load by providing balance between the exploitation and exploration.	Computational speed can be improved.
Gowrishankar [10]	Basic Artificial Bee Colony BABC algorithm and modified Artificial Bee Colony algorithm	Framed an improved objective function for the reduction of the fuel consumption and emission.	The algorithm becomes stuck in local optima and may not achieve the global optimal solution.
Shuo Zhang & Rui Xiong(2015) [19]	Dynamic Programming (DP) method	Minimization of fuel usage	It is unable to guarantee optimality of the cost function (fuel consumption) and SOC limit constraints.
Namwook, K, Sukwon, C & Huei, P 2011 [20]	Pontryagins Minimum Principle(PMP) and Dynamic Programming (DP) method	Described the global optimality of HEV fuel optimization from a mathematical viewpoint	The car's emission levels are not compared to any global standard to demonstrate that the optimized vehicle is within practical limitations.
Proposed method	Powell's Based Artificial Bee Colony Approach	Designed an improved objective function for the reduction of fuel consumption and emission.	Still the searchability and convergence speed of the search equation can be improved

The technique for the optimal design is either for the component sizing or the control strategy, while another parameter is maintained fixed, which is a key similarity across all the studies described above. Additionally, in some research, it is either the emission parameter or fuel usage. Even if both are considered, the energy redistribution and efficiency change of the important vehicle components before and after optimization is still not considered. However, in a real-world situation, a vehicle's component sizing and control technique interact to provide the best performance possible. The main innovation idea of the proposed work is to achieve a more optimized design for a hybrid electric vehicle, one that combines optimal component sizing with decreased fuel consumption and emissions. It becomes essential to optimize component sizes and control strategy parameters at the same time. To optimize the HEV under consideration and enhance its performance in terms of component size, fuel consumption, and emissions, the design optimization strategy looks for an efficient technique. The key objective function of this paper is to analyze a parallel HEV design extensively and to optimize its design, to obtain maximum vehicle performance in terms of fuel consumption, emissions, and better energy distribution, compared to its initial design.

## 2 Materials and methods

### 2.1 Simulation tool- ADVISOR

Advanced Vehicle Simulator (ADVISOR) [23] has been developed in November 1994 by NREL lab at USA. The main purpose of the tool development was to help Hybrid Electric Vehicle (HEV) program management from the U.S. Department of Energy's (DOE) and to handle its technical challenges to design high-efficiency HEVs, this tool works on the MATLAB platform. ADVISOR estimates fuel economy, emissions on given cycles, the drivetrain component performance as well as capability due to maximum-effort acceleration.

Some critical goals of ADVISOR tool include.

- Accurate, which allows meaningful comparison of various configurations of the drivetrain
- Fast, which allows design space investigations and high-speed analysis of vehicles, such as optimization and multi-dimensional study of various vehicle parameters
- Flexible, which allows the user to evaluate vehicles with combinations of components and various control strategies

### 2.2 HEV vehicle design parameters

For the analysis, an electric vehicle with parallel hybrid powertrain is considered with the battery as a power source for gasoline engine. With the ADVISOR tool the vehicle is modelled and with the help of MATLAB (Mathworks. 2010) [24] the vehicle system parameters are fine tuned. By feeding the vehicle parameters listed in Table 2 to the heuristic approach, the fuel consumption and emission values are optimized.

**Table 2:** Vehicle parameters.

Vehicle Parameter	Description
Engine Capacity	Geo 1.0L
Engine Power	41Kilo Watts
Engine Type	Spark Ignition (SI)
Power Train	Parallel hybrid
Motor	Westinghouse, 75 kilo Watts, Induction AC motor
Efficiency of the Motor	92 %
Transmission	Five speed manual
Battery	12 V 26 A Hour Capacity
Battery type	Valve Regulated Lead Acid
Mass of the vehicle	592 Kilo grams
Efficiency of the Fuel converter	34 %
Exhaust after treat	Spark Ignition (SI) based Exhaust system
Frontal area (Af)	2.0 meter2
Type of drive	Front wheel drive
Coefficient of drag (Cd)	0.335
Car wheel radius (Rw)	0.282 m

#### 2.2.1 HEV power train modelling

Getting better performance in terms of fuel economy and emission of a HEV from powertrain design perspective is always challenging. With this type of subsystem modelling the better performance of the vehicle is achieved, which will also affect the emission and fuel economy.

#### 2.2.2 Engine modelling

The purpose of emission and fuel consumption reduction is achieved with appropriate engine modelling.

#### 2.2.3 Electric motor modelling

While selecting the motor the key parameter to be considered for HEV includes power, speed-torque characteristics, and the efficiency when coupled with battery and the engine.

#### 2.2.4 Driving cycle

A driving cycle is a progression of data points that plot the vehicle's speed against time. Driving cycles are established to evaluate vehicle performance, often in terms of fuel consumption and pollutants. Another important application of the driving cycle is vehicle simulation. Because the vehicle under consideration is a light duty passenger automobile, the basis driving cycles are the UDDS, FTP, and ECE-EUDC. In addition, UDDS and FTP are US cycles, whereas ECE-EUDC is a European Union term. The UDDS depicts the city driving conditions of a passenger car vehicle. It is a city route with 17 stops covering a distance of 7.5 miles. The average and the greatest speeds are 19.6 miles per hour and 56.7 miles per hour. The idle time and the total cycle time are 259 s and 1369 s correspondingly.

The FTP is like the UDDS with the addition of the first 505 s of an extra UDDS cycle. It is a city route that covers 11.04 miles. The average and maximum speeds are 21.2 and 56.7 miles per hour. The cycle has a total duration of 1874 s. ECE-EUDC (also known as NEDC) driving cycle consists of four repeated ECE segments without interruption, ensued by a single EUDC phase. The cycle lasts 1180 s in total, with ECE segments accounting for around 780 s and EUDC segments accounting for the final 400 s. This driving cycle's average speed would be 20.73 mph. These vehicle driving cycles are taken into account since they encompass the bulk of the various driving circumstances that a vehicle might encounter during its use.

### 2.3 Multi constraint optimization of the hybrid electric vehicles

Multi-constraint optimization, commonly known as Constrained Multi-Objective Optimization (CMOP), is a method for optimizing many conflicting objectives while satisfying certain constraints. The purpose of multi-constraint optimization is to identify a set of solutions that balance convergence, diversity, and practicality. The objective of the proposed approach is to design a HEV with reduced fuel consumption and emissions. M. Montazeri-Gh and A, Poursamad [25] did an analysis on torque speed characteristics of an IC engine, shows that the operating point for the low Fuel consumption and low emissions are different. If the equal weightage is considered for all the parameters (Fuel consumption and emissions), it cannot represent a practical vehicle design. So we have assigned different weighting factors for the emission and fuel consumption. The vehicle parameters are optimized with 4 different Objective Function (Obj Fn), in that Obj Fn 1,2 have 50 % weightage for the three different Emissions like Hydrocarbons (HC), Carbon MonOxide (CO), Oxides of Nitrogen (NOx), the remaining 50 % weightage is given to the fuel utilization. The Obj Fn 3, 4 have 30 % weightage is allotted for the fuel usage and remaining 70 % weightage is given for the emission. These different weightage factors for different driving cycles are considered for this analysis.

(1) $$\mathrm{Min}\;F\left(x\right)=b_{1}\text{FC}+b_{2}\text{CO}+b_{3}\text{NOX}+b_{4}\text{HC}$$

Where F(x) is the function to be optimized,

b_1_ to b_4_ are weighting factors for various metrics,

(FC) and emissions (CO, NOX, and HC), considered in the objective function, subject to PNGV constraints.

Furthermore, the considered system is relevant to the following environmental circumstances mentioned in Equations (2), (3), (4):

The engine can only yield power:

(2) $$P_{\text{eng}}>\;0$$

The engine's maximum power rating is quoted as:

(3) $$0<P_{\text{engine}}<P_{\text{engine}\_\max\_\text{power}}$$

To function as a charge-sustaining control system, the State of Charge (SOC) of the battery should always be within the stated minimum and maximum limits:

(4) $${\text{SOC}}_{\text{low}}<{\text{SOC}}_{\text{bat}}<{\text{SOC}}_{\text{high}}$$

Within the PNGV constraints, the vehicle design parameters has been optimized. The search equation is modified to get optimal searchability and reduces the error and computation time.

#### 2.3.1 Modified Artificial Bee Colony (MABC) based optimization

Shivappriya S N [26] shows the control strategical parameters of the parallel HEV with EACS using Modified ABC combined SQP approach. The same vehicle parameters are used in this proposed work, for better vehicle design, a new optimization approach called Powell's ABC is introduced. Gowrishankar T [10] elaborated how the BABC is very good at exploration, whereas it is poor at exploitation, and also how this problem is solved with the Modified ABC (MABC) algorithm. Zhu & Kwong [27] found a global best solution for candidate search; this concept is utilized in the MABC algorithm as global best guided Algorithm. With this equation (5) the global best solution is identified

(5) $$S_{i,j}=D_{i,j}+\Phi_{i,j}\left(D_{i,j}-D_{k,j}\right)+\Psi_{i,j}\left(D_{newj}-D_{i,j}\right)$$

S _*i*,*j*_ is the new neighbouring source of food,

D_new*j*_ is the overall best solution of *j*th element,

Ψ _*i*,*j*_ generates random number between 0 and 1.5,

Φ _*i*,*j*_ is a random number generator within −1 to 1,

*j*∈ {1, 2, …, *n*} is a at random selected index.

Ψ_*i*,j_ (D_new*j*_ −D _*i*,j_) is the Gbest term added in addition to basic ABC.

Good optimization performance can be achieved by balancing exploitation and exploration. MABC has some disadvantages like slow convergence and weak local search. Hence the paper is proceeded with Powell's method-based ABC, which has a strong ability of local search. Gao & Liu [28] implemented opposition-based learning with chaotic systems method for improving global convergence speed. The details of Powell's method-based ABC are described in the next section.

#### 2.3.2 Powell's method based artificial bee colony (PABC) optimization

The method for artificial bee colonies (ABCs) outperforms conventional population-based algorithms. ABC, on the other hand, still has an issue with its solution search equation, which excels in exploration but struggles with exploitation. The Powell's ABC algorithm balances exploration and exploitation, allowing it to search widely across the solution space while refining promising areas. This helps avoid local optima, a common issue in optimization. It ABC mimics the foraging behaviour of bees, leveraging collective intelligence to explore and exploit the solution space effectively. This natural approach enhances the ability to find optimal or near-optimal solutions. To solve this problematic issue, we first offer a modified search equation that is used in the onlookers phase to develop a potential solution and increase ABC's search ability [29],no other additional facilities are required.

Alkin Yurtkuran & Erdal Emel [30] used the random key for searching the minimax location-allocation (p center) problem effectively. The proposed approach focuses the global best solution with the enhanced convergence speed by using the below equation (6)

(6) $$S_{i,j}=D_{k,j}+rand\left(0,1\right)\left(D_{best,j}-D_{k,j}\right)$$

rand function creates a random number between 0 and 1,

k is an integer and is different from *i*, and D_best_ is the best solution in the recent population.

Powell proposed this algorithm for finding a local least of a function, which is non differentiable, and could not take derivatives [31].

A set of initial search vectors is also passed in by the caller. N Search Vectors (SV) (for example, SV_1_,SV_2_, ….SV_N_) are typically provided in, which are essentially the normal aligned to each axis. To minimize the function, a bidirectional search is performed along each search vector in turn.

To execute bi-directional line searches along each search vector, utilize the golden section search or Brent's technique [32].

Bi-directional line search *minima:{r*_*0*_*+ μ*_*1*_*SV*_*1*_*, r*_*0*_*+* $\sum_{i=1}^{2}{\mu_{i}SV}_{2}$*, r*_*0*_*+* $\sum_{i=1}^{N}{\mu_{i}SV}_{i}\text{,}$

r_0_ is the initial starting point during bi-directional search along SV_i_.

The new position (r_1_) can then be represented as r_0_+ μ_1_SV_1_.The new transposition vector is considered as a new search vector [33].

Most successful *index = argmax(*$\max_{1\leq i\leq N}\left\|\mu_{i}\right\|\left\|{SV}_{i}\right\|)$ ,

new collection of N search vectors*: { SV*_*1*_*, ….SV*_*index-1*_*,SV*_*index+1*_*, ….SV*_*N*_*,* $\sum_{i=1}^{N}{\mu_{i}SV}_{i}\;\}$

The algorithm iterates indefinitely until no substantial improvement is achieved. Because no derivatives are necessary, the method is useful for calculating the local minimum of a continuous but complex function, particularly one lacking an underlying mathematical definition. The complexity stems from the linear searches along the search vectors, which can be completed using Brent's technique.

Minimization of the function takes place in this method because of two directional searches. The new search position can then be expressed as a linear combination of the search vectors. The search vector list adds the new displacement vector at the end of the vector list and becomes a new search vector. For the Meantime, the search vector which was most successful in contributing to a new path is deleted from the search vector list. The process is repeated or iterated an arbitrary number of times until the new feasible solution converges to a value, where no further significant improvement is made.

**Fig. 1:**
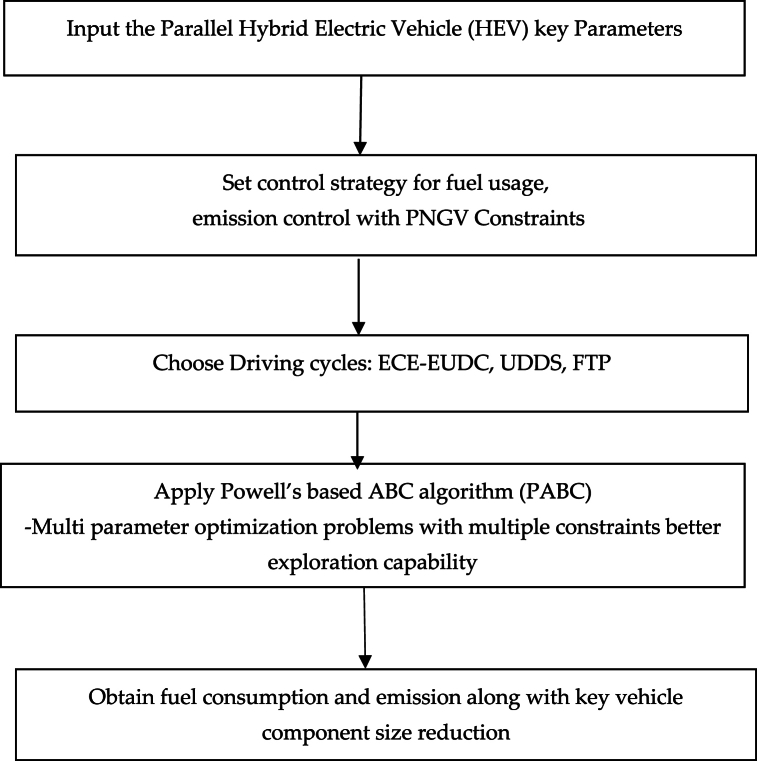
Flow chart of the Proposed Approach.

With Powell's method combined with ABC, they have complementary advantages with improved local searchability, and convergence speed of the ABC can be improved, and this results in a better optimized solution with least convergence time. The flow chart of the proposed approach is shown in Fig. 1.

Step 1 Input the Parallel Hybrid Electric Vehicle (HEV) key parameters,
Step 2 Fixing the control strategy for fuel usage, emission control with PNGV Constraints
Step 3 Selection of the driving cycle.
Step 4 Apply the vehicle parameter optimization through the PABC approach, Multi parameter optimization problems with multiple constraints with better exploration capability.
Step 5 Obtain the fuel consumption and emission along with key vehicle component size reduction.

## 3 Results and discussion

With the ADVISOR tool hybrid modelling good acceleration and high-speed execution is achieved. We have used the three base driving cycle ECE EUDC, UDDS, FTP with the PNGV constraints [34]. The proposed work provides a balance between the energy management, component size and control strategy, which affect one another. It is necessary to concurrently optimize the energy management, control strategy and component size to have efficient vehicle operation.

### 3.1 PABC ECE-EUDC driving cycle results

With the Powell's algorithm, the optimal solution is identified for all the driving cycles considered and results for.

ECE-EUDC driving cycle with the variables, constraints and optimized values are listed in Table 3. The below figures

**Table 3:** ECE-EUDC driving cycle results with PABC.

Description		Base Value	Obj Fn 1	Obj Fn 2	Obj Fn 3	Obj Fn 4
Vehicle Parameter	Fuel Convertor torque scale	1.349	1.500	1.262	1.385	1.200
	Motor Controller torque scale	1.182	0.746	1.133	0.966	0.870
	Number of battery modules	30.000	23.000	22.000	21.000	25.000
	Electric launch Speed low	3.000	4.000	1.000	4.000	3.126
	Electric launch Speed high	20.000	32.000	15.000	31.000	16.902
	Minimum torque fraction	0.218	0.212	0.361	0.317	0.359
	Off torque fraction	0.137	0.095	0.139	0.050	0.183
	SOC low	0.567	0.487	0.500	0.507	0.516
	SOC high	0.695	0.776	0.780	0.632	0.845
	Charge Torque	31.000	15.000	22.000	31.000	5.199
Vehicle Performance	Fuel Consumption (miles per gallon)	28.600	31.469	31.464	31.251	28.913
	Hydrocarbons (grams/mile)	0.768	0.828	0.732	0.803	0.715
	Carbon MonOxide (grams/mile)	3.157	2.693	2.81	2.74	2.576
	Oxides of Nitrogen (grams/mile)	0.495	0.469	0.488	0.503	0.479
	Overall System Efficiency	10.4 %	10.6 %	11.0 %	11.1 %	11.1 %
	Fuel Converter	24 %	22 %	24 %	23 %	24 %
	Torque coupling	99 %	100 %	100 %	100 %	100 %
	Energy storage	87 %	87 %	86 %	86 %	87 %
	Motor(Power mode)	61 %	64 %	71 %	76 %	48 %
	Motor(Regen mode)	55 %	57 %	65 %	65 %	50 %

show the change in performance of the vehicle after its design optimization with weighting factors as per Obj Fn 1.

**Fig. 2:**
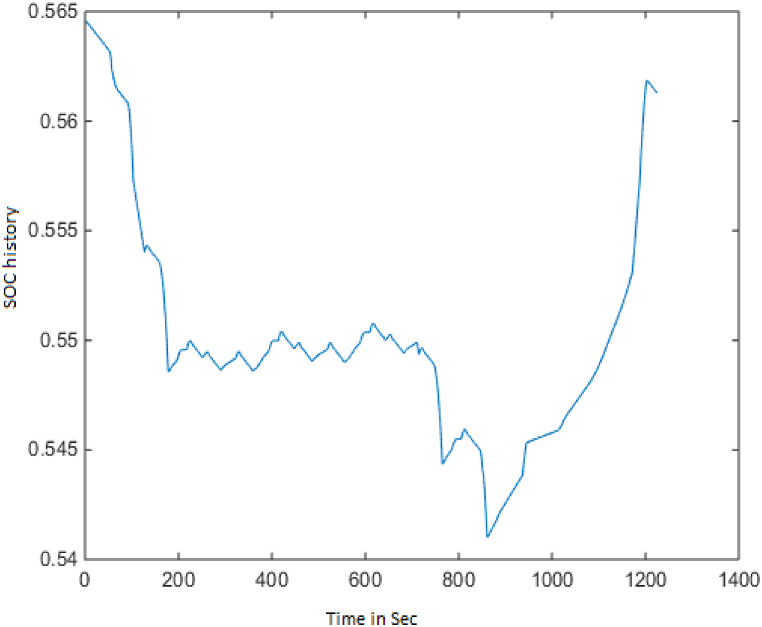
SOC history.

ECE-EUDC driving cycle length is 1200 s, and Fig. 2 shows the variance of the SOC battery

approximately near to 56 %.

**Fig. 3:**
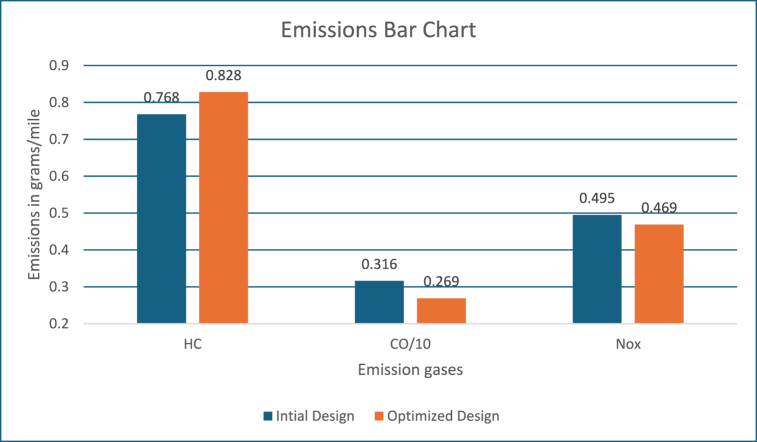
Emissions bar chart.

Fig. 3 shows the emission values in grams/mile. These figures show, the engine system energy utilization is 20000 kJ with the power mode and energy utilized is close to 800 kJ by the motor controller during regeneration mode, which is used for recharging the battery as well as keeping it within the required SOC level. The fuel converter efficiency is close to 22 %, which is like the previous approach. Also, energy usage by braking components to support higher energy regeneration is around 500 kJ. The PNGV constraints and SoC requirements are met.

**Fig. 4:**
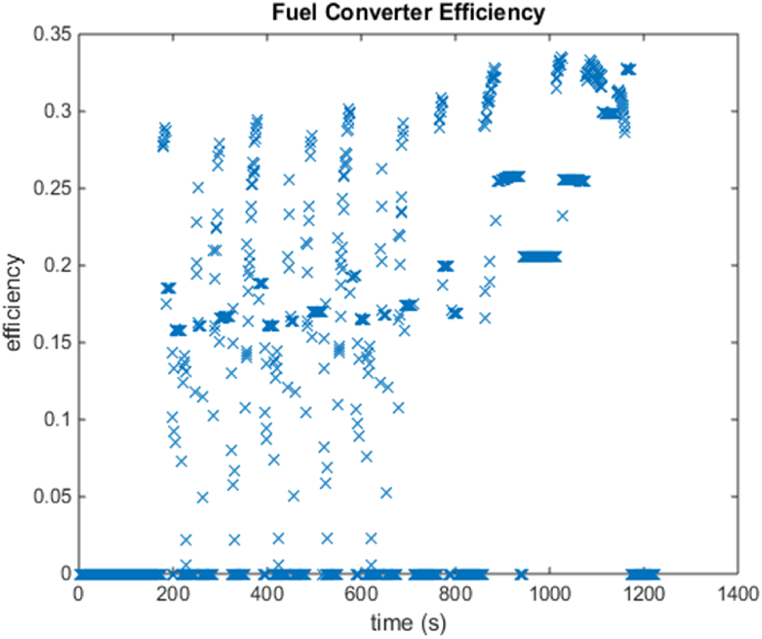
Engine system Efficiency.

**Fig. 5:**
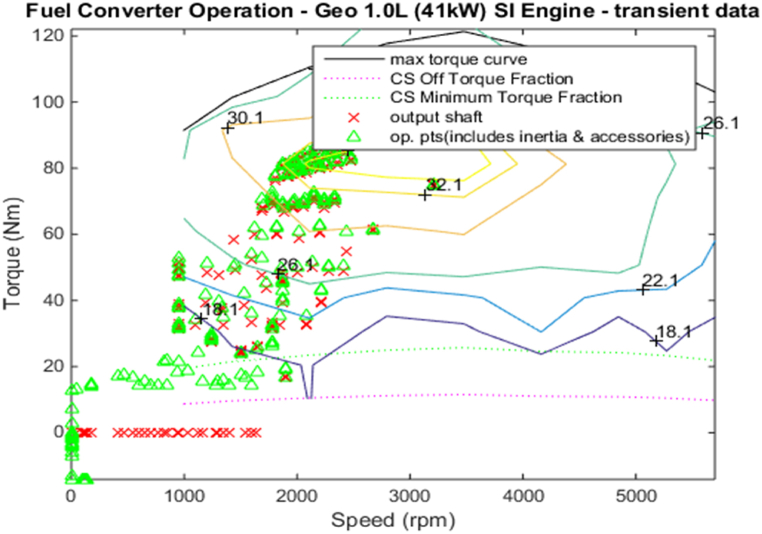
Engine system Operation.

**Fig. 6:**
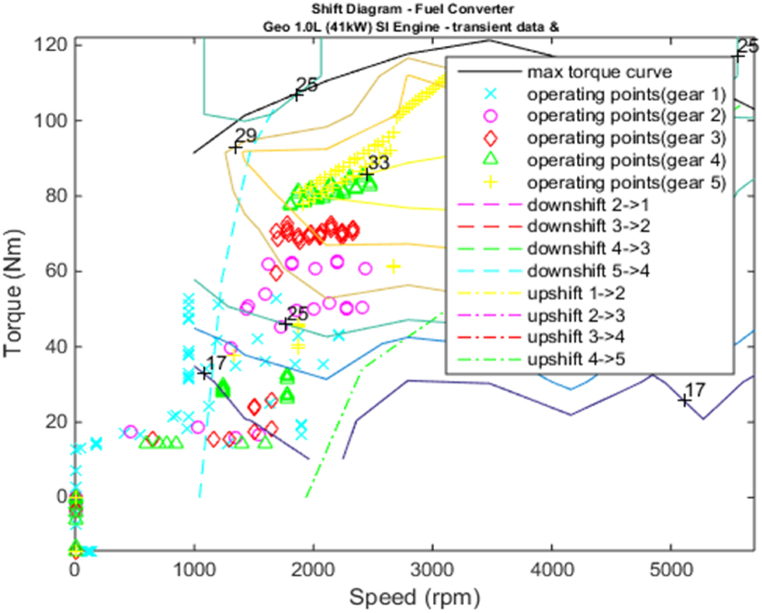
Gear shift diagram.

**Fig. 7:**
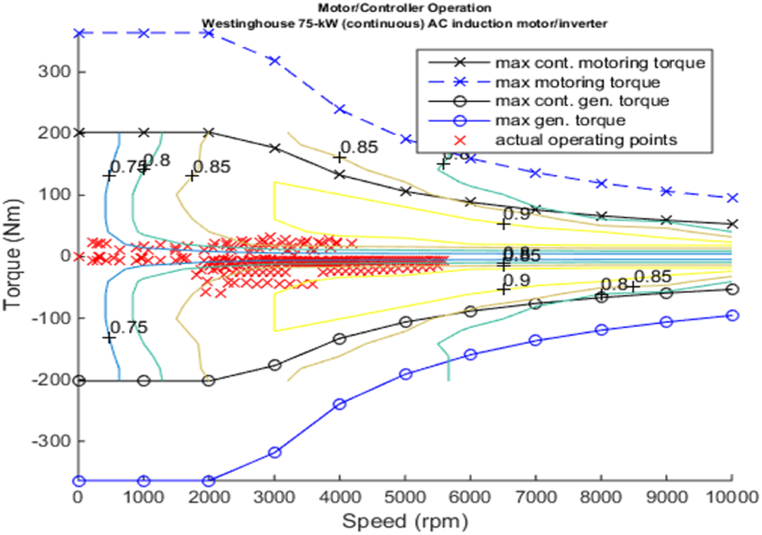
Motor torque-speed.

Fig. 4, Fig. 5 shows the efficiency of the engine system, and its operation, Gear Shift Diagram and electric motor torque-speed characteristic is shown in Fig. 6, Fig. 7.

#### 3.1.1 Inference on fuel usage for ECE-EUDC driving cycle

**Fig. 8:**
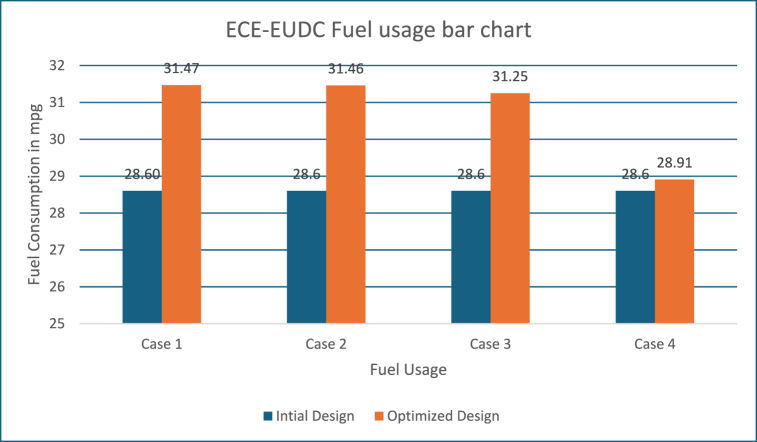
ECE-EUDC fuel usage.

Also, the improvement in fuel efficiency for all the four strategies with the driving cycle is given below in Fig. 8.

It is evident from the above Fig. 8 Higher weightage is fixed for FC in the Obj Fn 1 and 2, which results increase in FC when compared to other two strategies (Obj Fn 3 and 4).

### 3.2 PABC UDDS driving cycle results

With the Powell's algorithm, the optimal solution is identified for all the driving cycles considered and results for UDDS driving cycle with the variables, constraints and optimized values are listed in Table 4. The below figures show the change in performance of the vehicle after its design optimization with weighting factors as per Obj Fn 1.

**Table 4:** UDDS driving cycle results with PABC.

Description		Base Value	Obj Fn 1	Obj Fn 2	Obj Fn 3	Obj Fn 4
**Vehicle Parameter**	Fuel Convertor torque scale	1.349	1.245	1.245	1.245	1.245
	Motor Controller torque scale	1.182	0.738	0.947	1.082	1.133
	Number of battery modules	30.000	22.000	22.000	22.000	30.000
	Electric launch Speed low	3.000	1.000	1.000	1.000	1.000
	Electric launch Speed high	20.000	15.000	15.000	15.000	15.000
	Minimum torque fraction	0.218	0.361	0.361	0.344	0.278
	Off torque fraction	0.137	0.139	0.139	0.139	0.139
	SOC low	0.567	0.500	0.500	0.500	0.261
	SOC high	0.695	0.844	0.844	0.844	0.844
	Charge Torque	31.000	37.000	32.000	32.000	33.000
**Vehicle Performance**	Fuel Consumption (miles per gallon)	31.400	37.120	35.878	34.848	33.257
	Hydrocarbons (grams/mile)	0.737	0.666	0.671	0.676	0.681
	Carbon MonOxide (grams/mile)	4.833	2.743	2.903	3.174	3.768
	Oxides of Nitrogen(grams/mile)	0.557	0.507	0.520	0.530	0.539
	Overall System Efficiency	9.0 %	10.1 %	9.8 %	9.6 %	9.5 %
	Fuel Converter	27 %	26 %	26 %	26 %	27 %
	Torque coupling	100 %	100 %	100 %	100 %	100 %
	Energy storage	87 %	87 %	87 %	87 %	87 %
	Motor(Power mode)	65 %	68 %	61 %	57 %	56 %
	Motor(Regen mode)	65 %	69 %	63 %	60 %	60 %

**Fig. 9:**
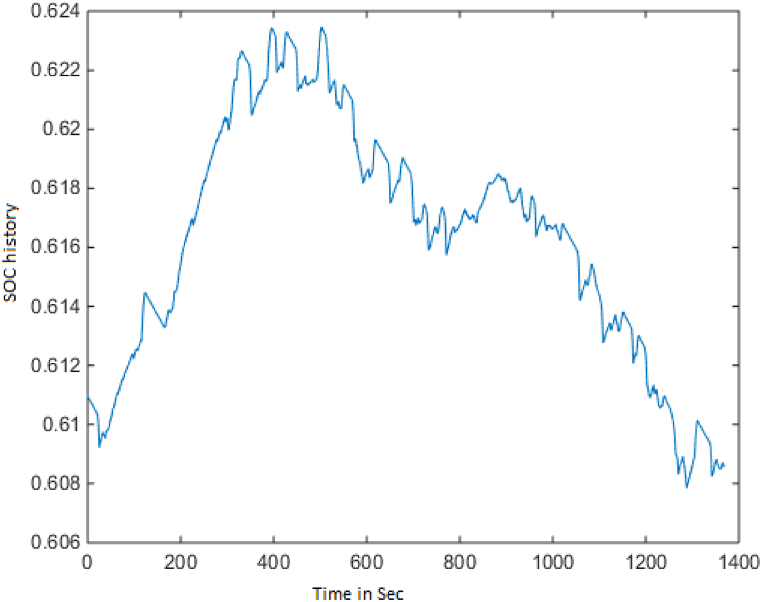
SOC history.

These figures show that the fuel converter consumes more energy during power mode, approximately 18000 kJ, and the motor controller consumes approximately 850 kJ during regeneration mode, in order to recharge the battery and keep it within the intended SOC level. The length of UDDS driving cycle is a 1369-s.And it can be observed that, while the fuel converter's efficiency is only about 26 %, as in the prior scenario, the energy loss has been reduced following optimization. In addition, brake components consume close to 900 kJ of energy in this mode, allowing for more energy regeneration. The PNGV limitations and the SoC requirements have been met. Fig. 9 depicts the variation in battery SOC with respect to this driving cycle, and it can be seen that the variation between the initial and final value of battery SOC is less than 0.5 %, with a value close to 61 %.

Fig. 11, Fig. 12, Fig. 13, Fig. 14 shows the engine system efficiency, operation, Gear Shift Diagram and also electric motor torque-speed characteristics.

**Fig. 10:**
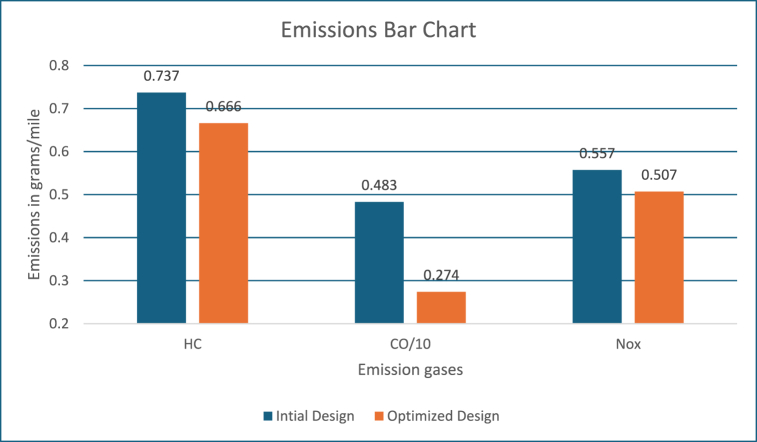
Emissions bar chart.

**Fig. 11:**
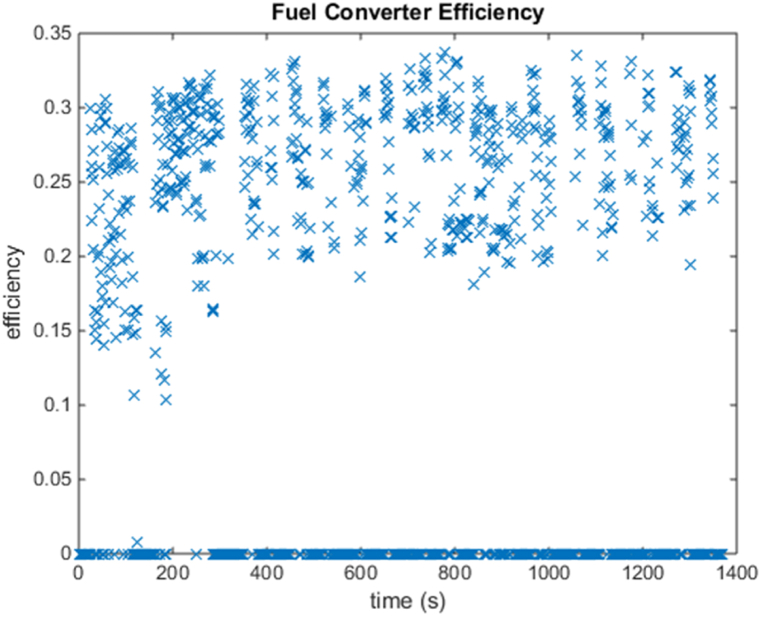
Engine system Efficiency.

**Fig. 12:**
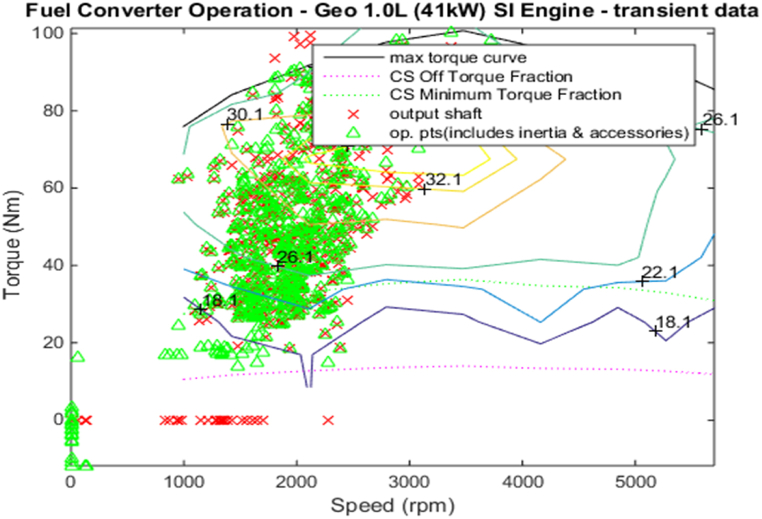
Engine system Operation.

**Fig. 13:**
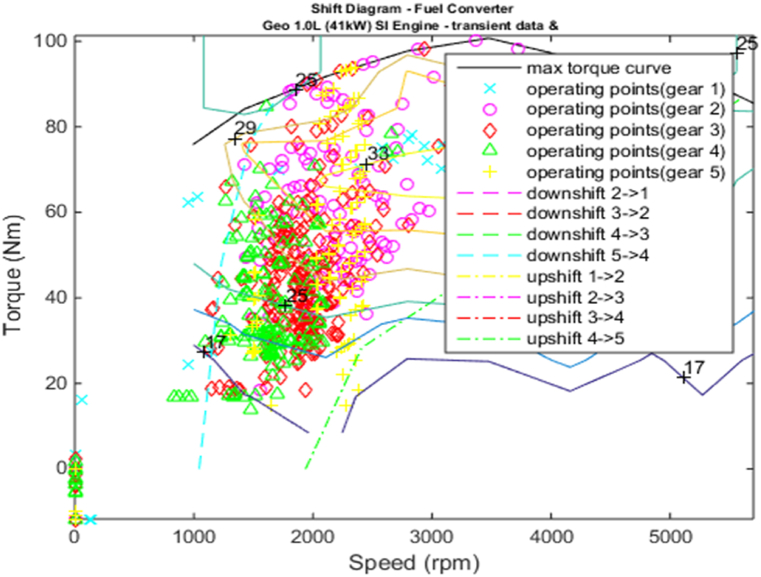
Gear shift diagram.

**Fig. 14:**
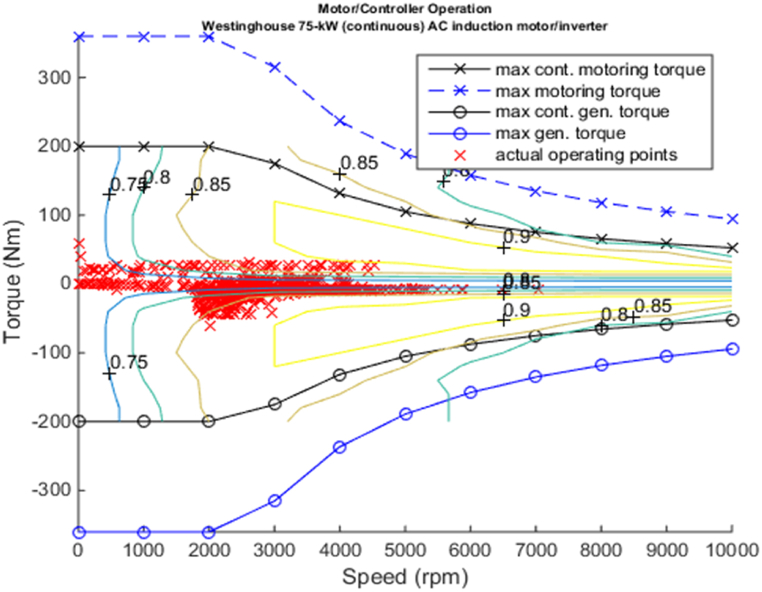
Motor torque-speed.

#### 3.2.1 Inference on fuel usage for UDDS driving cycle

**Fig. 15:**
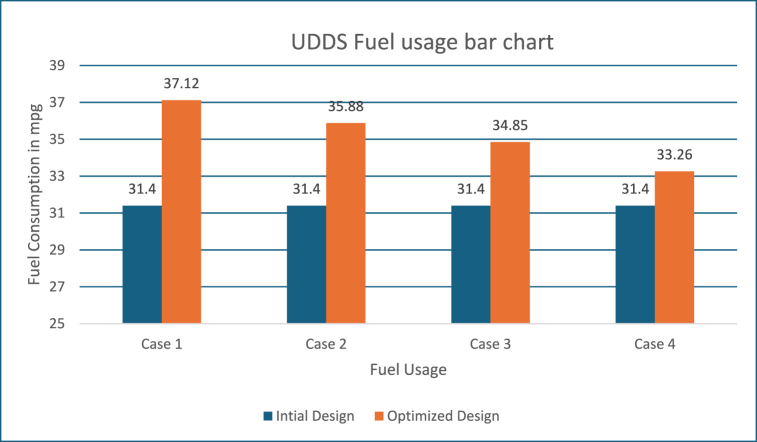
Udds fuel usage.

**Fig. 16:**
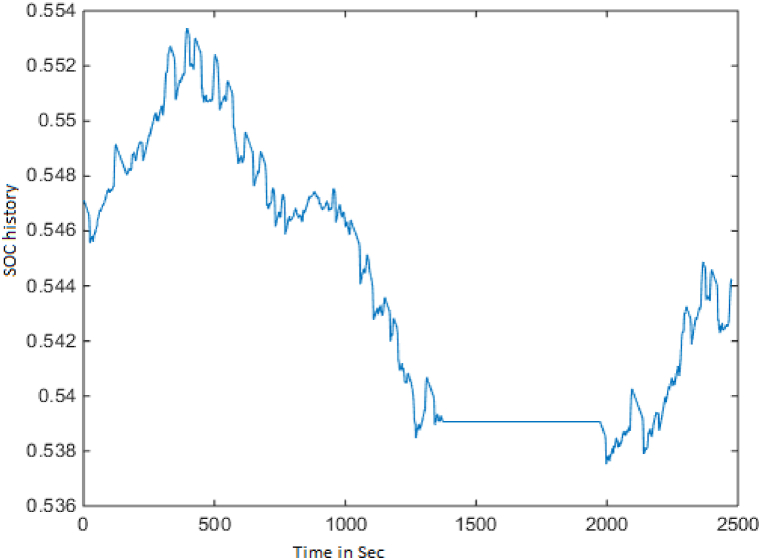
SOC history.

Also, the improvement in fuel efficiency for all the four strategies with the driving cycle is given below in Fig. 15. It is evident from the above Fig. 16, Higher weightage is fixed for FC in the Obj Fn 1 and 2, which results increase in FC when compared to other two strategies (Obj Fn 3 and 4). As mentioned earlier, with Powell's method combined with ABC, they have complementary advantages with improved local searchability, and convergence speed of the ABC can be improved and this result in a more optimized solution, but this does not show much efficient with respect to exploration.

### 3.3 PABC FTP driving cycle results

With the Powell's algorithm, the optimal solution is identified for all the driving cycles considered and results for FTP driving cycle with the variables, constraints and optimized values are listed in Table 5. The below figures show the change in performance of the vehicle after its design optimization with weighting factors as per Obj Fn 1. The length of the FTP driving cycle is 2500 s. Also, Fig. 16 shows the variance of the SOC battery approximately near to 54 % (see Table 6).

**Table 5:** FTP driving cycle results with PABC.

Description		Base Value	Obj Fn 1	Obj Fn 2	Obj Fn 3	Obj Fn 4
**Vehicle parameters**	Fuel Convertor torque scale	1.349	1.020	1.245	1.245	1.118
	Motor Controller torque scale	1.182	0.637	0.623	0.620	0.741
	Number of battery modules	30.000	24.000	22.000	27.000	24.738
	Electric launch Speed low	3.000	1.000	1.000	4.546	7.414
	Electric launch Speed high	20.000	17.000	15.000	15.000	27.000
	Minimum torque fraction	0.218	0.710	0.361	0.361	0.143
	Off torque fraction	0.137	0.163	0.139	0.139	0.128
	SOC low	0.567	0.443	0.500	0.500	0.426
	SOC high	0.695	0.795	0.831	0.844	0.859
	Charge Torque	31.000	29.000	5.000	5.000	6.000
**Vehicle performance**	Fuel Consumption (miles per gallon)	32.200	40.037	35.600	34.679	33.536
	Hydrocarbons (grams/mile)	0.564	0.432	0.507	0.509	0.489
	Carbon MonOxide (grams/mile)	3.244	3.009	1.781	1.885	2.130
	Oxides of Nitrogen (grams/mile)	0.471	0.383	0.400	0.405	0.400
	Overall System Efficiency	9.9 %	10.1 %	10.3 %	10.3 %	11.6 %
	Fuel Converter	27 %	24 %	24 %	24 %	27 %
	Torque coupling	100 %	100 %	100 %	100 %	100 %
	Energy storage	87 %	87 %	87 %	87 %	86 %
	Motor(Power mode)	66 %	63 %	62 %	44 %	71 %
	Motor(Regen mode)	64 %	61 %	62 %	60 %	70 %

**Table 6:** Optimization of Vehicle design parameters.

Driving cycle		Powells ABC Approach	
Vehicle design parameters	ECE-EUDC cycle	UDDS cycle	FTP cycle
Fuel efficiency	10.3 %	18.2 %	24.33 %
Emissions reduction	18.4 %	43.24 %,	45.09 %
Overall vehicle efficiency	11.1 %	10.1 %	11.6 %
Engine Torque scale	1.349 is reduced to 1.200	1.349 is reduced to 1.024	1.349 is reduced to 1.020
Motor torque scale	1.182 is reduced to 0.746	1.182 is reduced to 0.623	1.182 is reduced to 0.620
Number of battery modules	30 is reduced to 21	30 is reduced to 24	30 is reduced to 22

**Fig. 17:**
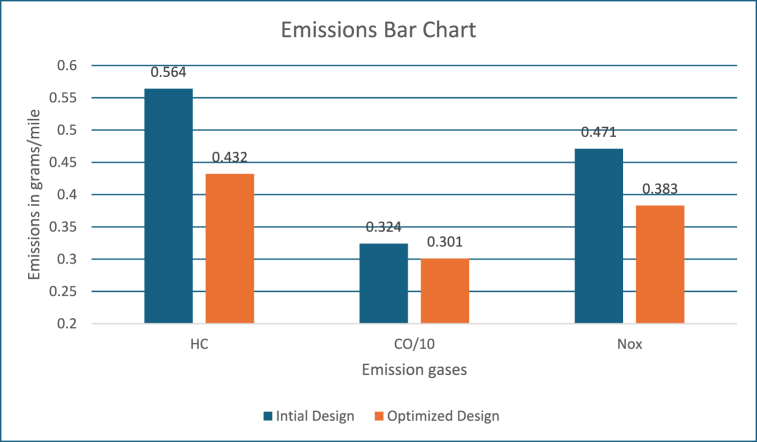
Emissions bar chart.

Fig. 17 shows the emission values in grams/mile. The engine system energy utilization is 24000 kJ with the power mode and energy utilized is close to 1100 kJ by the motor controller during regeneration mode, which is used for recharging the battery as well as keeping it within the required SOC level. The engine system efficiency is close to 24 %, which is like the previous approach. Also, energy usage by braking components to support higher energy regeneration is around 1250 kJ.

**Fig. 18:**
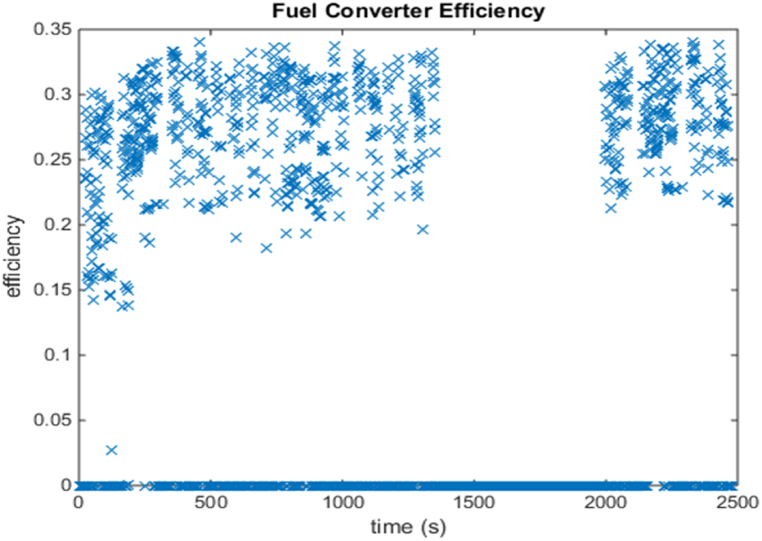
Engine system Efficiency.

**Fig. 19:**
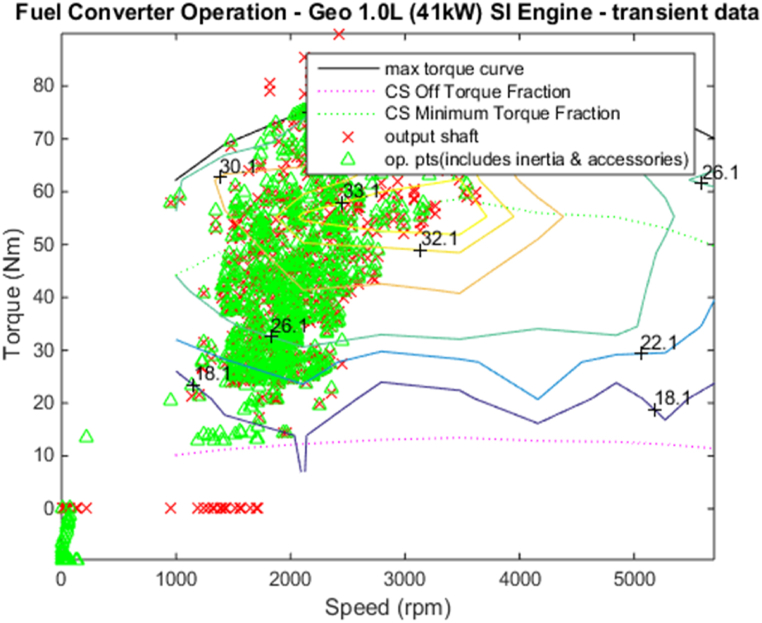
Engine system Operation.

**Fig. 20:**
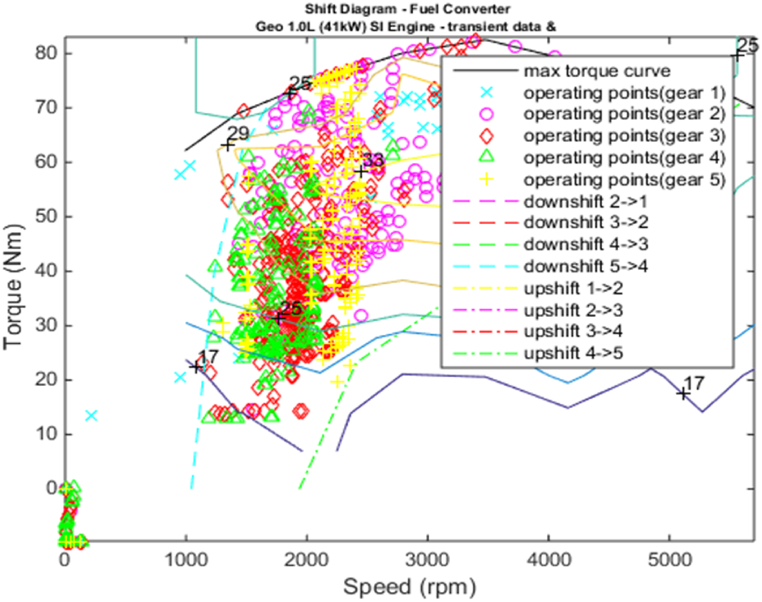
Gear shift diagram.

**Fig. 21:**
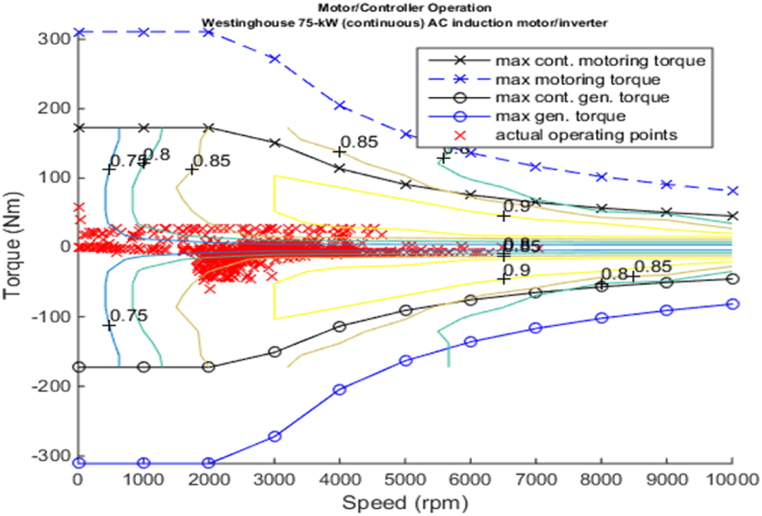
Motor torque-speed.

Fig. 18, Fig. 19 shows the efficiency of the engine system, and its operation, Gear Shift Diagram and electric motor torque-speed characteristic is shown in Fig. 20, Fig. 21.

#### 3.3.1 Inference on fuel usage for FTP driving cycle

**Fig. 22:**
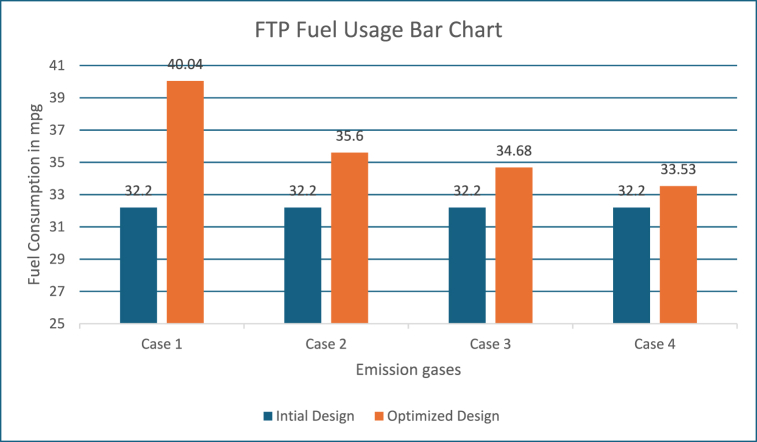
FTP fuel usage.

Also, the improvement in fuel efficiency for all the four strategies with the FTP driving cycle is given in Fig. 22. It is evident from Fig. 10, Higher weightage is fixed for FC in the Obj Fn 1 and 2, which results increase in FC when compared to other two strategies (Obj Fn 3 and 4).

For a similar vehicle configuration Gowrishankar T [10] did the state of art comparison of the BABC and MABC, which is taken into consideration for comparing our proposed work of PABC. There is a significant improvement when compared to MABC in the Fuel economy i.e. 19.3 % in terms of miles per gallon. The emission also decreased such as 40.33 % for HC, 4.62 % for CO and finally 25.84 % for NOx. In the similar way for the other three Obj Fns based upon the weighting factor the better results are achieved for the fuel economy and emission as discussed in the above sections. For the ECE-EUDC driving cycle, the initial efficiency was 10.4 % only, But after the optimization, there was a finite amount of decrease in fuel consumption and emissions, including the key components size reduction, which resulted in an improved overall vehicle efficiency up to 11.1 % and for UDDS, initial efficiency was 9.0 % and maximum efficiency of 10.1 % is achieved after optimization, while the vehicle parameters are still within the PNGV constraint limits. FTP driving cycle, the energy efficiency of the initial vehicle design was 9.9 % only and the maximum vehicle efficiency up to 11.6 % for the FTP driving cycle. PABC effectively optimizes the parameters to achieve effective energy efficiency in the circumstances like motor (regen mode), motor (power mode), energy storage, torque coupling, and fuel converter.

**Fig. 23:**
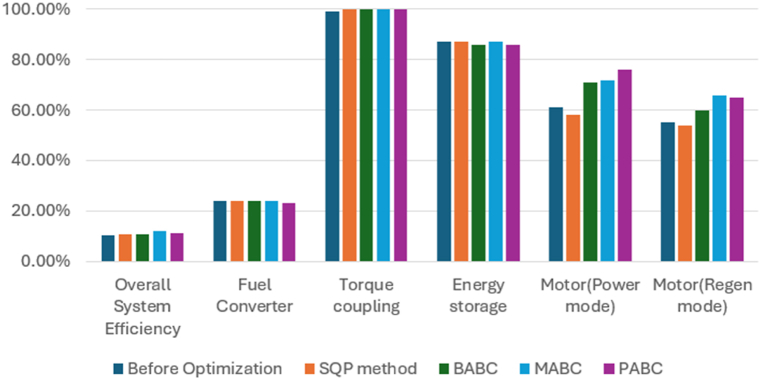
Performance comparison of Base value, SQP, BABC, MABC and PABC approaches for the ECE_EUDC driving cycles.

**Fig. 24:**
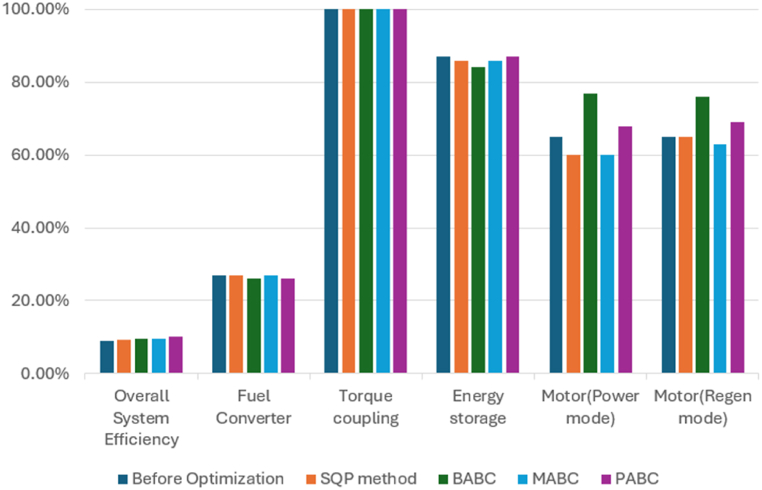
Performance comparison of Base value, SQP, BABC, MABC and PABC approaches for the UDDS driving cycles.

**Fig. 25:**
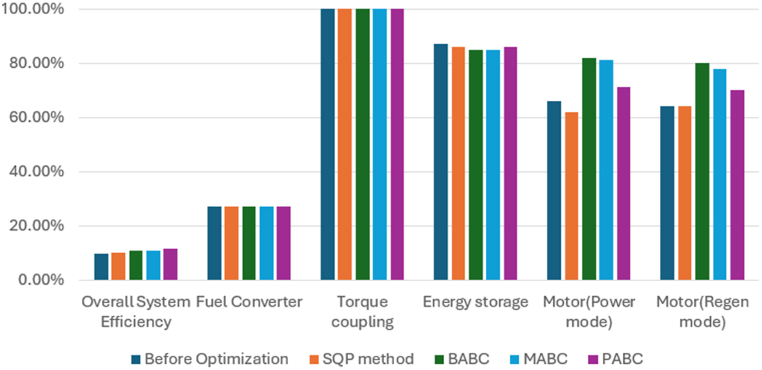
Performance comparison of Base value, SQP, BABC, MABC and PABC approaches for the FTP driving cycles.

Fig. 23, Fig. 24, Fig. 25 shows the performance comparison of Base value-before optimization and after optimization results of SQP, BABC, MABC and PABC approaches for the three driving cycles. The performance of Powell's ABC is better than the base design value of the vehicle parameters such as overall system efficiency, engine system -fuel convertor efficiency, gear system-torque coupling, battery -energy storage, motor (power) efficiency, motor (regeneration mode) efficiency. The comparisons reveal that the proposed algorithm outperforms all competitors on practically all test functions in terms of solution quality, global convergence speed, and robustness.

## 4 Conclusion

This work focuses on a detailed design analysis and validation of a parallel HEV, for maximizing energy efficiency, to minimize the fuel consumption and gas emissions like Hydrocarbons (HC), Carbon Monoxide (CO), Oxides of Nitrogen (NOx) along with optimizing the size of the key components of the vehicle using PABC method. The evaluation was carried out with three different driving cycles namely ECE-EUDC an, UDDS, FTP driving cycles. Powell's method is merged to ABC as a tool for local search in order to enhance the local search capability and convergence velocity of the algorithm.

The proposed method performs better than the existing methods such as the Sequential Quadratic Programming (SQP), Basic Artificial Bee Colony (BABC) and Modified Artificial Bee Colony (MABC) in-terms of overall vehicle energy efficiency, fuel economy as well as exhaust gas emission. With Powell's method combined with ABC, they have complementary advantages with improved local searchability, and convergence speed of the ABC can be improved, and this results in a more optimized solution.

Compared with the initial values, the proposed approach gives the improvement in the ECE-EUDC, FTP and UDDS driving cycle has been tabulated in Table 3, Table 4, Table 5. The quantitative result shows how the optimization approach used for the reduction of overall vehicle key component size. Even with reduced battery modules the SOC change is within 0.5 % tolerance is maintained for meeting the required vehicle performance. In most of the test cases the engine, ESS, motor component size is also reduced.

## 5 Future work

In the future work, the convergence speed of the algorithm used above can be improved by using new evolutionary algorithms. Also, there is a scope for improvement in the number of vehicle design variables including environmental parameters, as this would result in performance output equivalent to real-world design, but this has requirement of complex optimization algorithms that could handle multi-dimensional data of the vehicle considered for the design. Also, the same approach can be extended to design high-performance vehicle such as racing vehicles, plug in hybrid vehicles, series HEV and fully electric vehicle.

## CRediT authorship contribution statement

**S.N. Shivappriya:** Formal analysis. **T. Gowrishankar:** Conceptualization. **Gabriel Stoian:** Methodology. **J. Anitha:** Investigation. **D Jude Hemanth:** Formal analysis.

## Data availability

MATLAB toolbox and diesel net website data are used to support the findings of this study and are included within the article.

## Funding statement

This research received no external funding.

## Declaration of competing interest

The authors declare that they have no known competing financial interests or personal relationships that could have appeared to influence the work reported in this paper.
